# The Dilemma of Inclusive Education: Inclusion for Some or Inclusion for All

**DOI:** 10.3389/fpsyg.2021.633066

**Published:** 2021-09-10

**Authors:** Äli Leijen, Francesco Arcidiacono, Aleksandar Baucal

**Affiliations:** ^1^Institute of Education, University of Tartu, Tartu, Estonia; ^2^Research Department, Haute Ecole Pédagogique BEJUNE, Biel/Bienne, Switzerland; ^3^Faculty of Philosophy, University of Belgrade, Belgrade, Serbia

**Keywords:** inclusive education, special education, public discourse, argumentum model of topics, dialogue

## Abstract

In this paper, we intend to consider different understandings of inclusive education that frame current public and professional debates as well as policies and practices. We analyze two – somewhat opposing – discourses regarding inclusive education, namely, the “inclusion for some” – which represents the idea that children with special needs have a right to the highest quality education which can be delivered by specially trained staff, and the “inclusion for all” – which represents the idea that all children regarding their diverse needs should have the opportunity to learn together. To put the two discourses in a dialogical relation, we have reconstructed the inferential configurations of the arguments of each narrative to identify how the two definitions contribute to position children with and without special needs and their teachers. The results show the possibilities to bridge the two narratives, with respect to the voices they promote or silence, the power relations they constitute, and the values and practices they enact or prevent.

## Introduction

Inspired by social justice ideas, the Convention on the Rights of the Child (UN, 1989) and the Salamanca Statement (UNESCO, 1994), many European countries have developed policies and implemented practices to promote inclusive education (Arcidiacono and Baucal, 2020; Nelis and Pedaste, 2020). Consequently, more children with special education needs^1^ are nowadays learning with their peers in mainstream schools and the number of special schools has decreased. Although this is a trend in different countries in Europe and in the Global North, there are several challenges. Most notably, there is still no clear understanding of inclusive education. Researchers, policy makers, and teacher educators have diverse understandings (Haug, 2017; Van Mieghem et al., 2018; Kivirand et al., 2020), which range from the idea that special education is itself a form of inclusive education, to the observation that all children are, for the majority, learning together in an inclusive setting (Ainscow and Miles, 2008; Hornby, 2015; Kivirand et al., 2020). Magnússon (2019) has concluded that the “implementations, interpretations and definitions of the concept vary greatly both in research and in practice, between countries and even within them” (*ibid*, p. 678).

These different discourses are present in several societies, but the debates are more heated in contexts which more recently have started to implement inclusive education practices, such as Eastern Europe and former Soviet countries (Florian and Becirevic, 2011; Stepaniuk, 2019). One of the reasons for so many challenges in the latter context is the past experience of a strongly segregated educational system. This historical context is illuminated in the views of teachers, parents, and the general public.

In this paper, we will analyze two – somewhat opposing – discourses regarding inclusive education encapsulating two positions that are in the core of many current debates about inclusive education. The first one (“inclusion for some”) represents the idea that children with special needs have a right to highest quality education which can be best delivered by specially trained staff in a specialized and often segregated environment, while the second one (“inclusion for all”) represents the idea that all children regarding their diverse needs should have the equal opportunity to learn together in a regular education setting.

In this paper, we are going to put the two discourses in a dialogical relation. Through an argumentative analysis based on the reconstruction of the inferential configurations of arguments, we intend to identify how the two definitions contribute to position children (with and without special needs) and teachers, whose voices they promote and whose voices are silenced, what power relations they constitute, and what values and practices they enact or prevent. The possibility to map out the reasoning beyond these arguments is discussed as the starting point for bridging the existing conceptions about inclusive education. Prior to introducing the two narratives, we introduce briefly the background of inclusive education in Estonia which forms the context of the current study.

## Inclusive Education in Estonia

Similarly to many Eastern European countries, Estonia has a long special education tradition, which is influencing acceptance of the principles and the actual practices of inclusive education. These principles have been established at the legislative level in Estonia since 2010, most notably the law states that students with special needs have the right for studying in their schools of residence with their peers (Basic Schools and Upper Secondary Schools Act, 2010, 2019). In accord with the changes in the legislative framework, the number of pupils with special educational needs in mainstream schools has increased; however, another phenomenon has appeared – the number of students enrolled in special classes in mainstream schools has also increased (Räis et al., 2016). These special classes are often taught by teachers of special education and not by regular teachers. Although many school leaders understand the need for inclusive education, their main concern is a lack of availability of support specialists – including special needs teachers, speech therapists, and phycologists (Räis and Sõmer, 2016). Although the expertise of support specialists is highly valued in Estonian schools and kindergartens, more and more teachers have recognized the importance of their own professional development related to supporting diverse learners. For example, the comparison of TALIS 2013 and TALIS 2018 survey data (OECD, 2020) showed that teachers’ participation in professional development activities related to supporting diverse learners has significantly increased in Estonia and at the same time teachers indicated that training in this area is for them still the largest need for professional development. Consequently, diverse in-service training courses are available for teachers. An analysis of the course content at one of the major universities in Estonia providing teacher education showed that the core content of these courses has tended to focus on didactical methods of teaching students with special educational needs rather than on strategies of inclusive pedagogy. However, more recent in-service courses have emphasized social justice, possibilities for participation, and inclusive pedagogies as well (e.g., Kivirand et al., 2021). This brief overview illuminates that very different perspectives and practices are present in Estonia. We will explore these in more detail in the next sections.

## Two Discourses of Inclusive Education

### Inclusion for Some

There have been several articles published in 2020 in Estonian national newspapers arguing that inclusive education is a dream or ideology that does not take into account actual circumstances of reality. In one of such articles (Ehala, 2020), a university professor, who regularly writes about education, cites a recent study conducted in Estonia on the added value of education on children’s cognitive abilities. The study showed that 80% of the children’s knowledge and skills can be explained by individual abilities and home background, and only 20% by the influence of school. The professor argued that children with physical disabilities could be included, but it is problematic to include children who have been raised according to very different principles or who have significant cognitive disabilities. He specified that inclusive education would only be possible in societies which are very homogeneous, most importantly regarding child raising practices and family values. This would result in a situation where there are few differences between children’s behaviors and are used for similar norms and regulations. He pointed out as: “Inclusive education is a mirage created by our sense of justice, but its implementation puts young people in a learning environment that is not in line with their home preparation and developmental needs. They are just too special and different so that everyone could learn together in a way that no one suffers.” He concluded that we simply need different kinds of environments for different children.

Many of these ideas are also pointed out by some teachers. In 2019, a new educational strategy was prepared for Estonia and in this process, several meetings were held in different places across the country. Many teachers were critical regarding the recent policy reform related to inclusive education. On the one hand, teachers are concerned about the learning process and outcomes of the regular children and, on the other hand, their own preparation to support students with special needs. Working with special needs students requires specialized knowledge and expert skills, which many teachers simply do not have. Similar to these views, a group of master students wrote an article in a national newspaper in June 2020 (Kupper et al., 2020) where they stated that although they support the idea of inclusive education, it is only justified if it is carefully organized and sufficient support is available. They also added that inclusive education is certainly not suitable for students with more severe special needs. They point out as: “Inclusion may not be effective in case the teacher does not receive enough support and guidance regarding how to work with a special needs student and the rest of the class at the same time. If, figuratively speaking, the teacher’s strength does not overcome the situation, then the increase in behavioral problems, drop-out rates and developmental delays are real dangers.”

Moreover, this article also shed light into the perspective of parents of special needs students. They argued as: “A familiar and close-to-home school with a teacher assistant or support specialist does not outweigh the assurance that the child’s safety and well-being is guaranteed throughout the day and is cared for by a sufficient number of professionals.” Moreover, “Studying at a school close to home may not always be possible if the child needs a much more complex service due to his or her situation, including, for example, special therapies and additional activities. If such a solution is not offered during the school day, parents must find the time and opportunity, usually at the expense of working hours, to provide the necessary service to the child. Thus, the difficulty of the whole situation lies with the parents, who, despite the child’s special needs, must be able to maintain optimism, offer equal care and love to the other children of the family in other words, try to live as normal a life as possible while maintaining the ability to work, good relations with the employer and income and one’s own emotional balance.”

In brief, all these perspectives argue that the development of different students will benefit from specialized learning environments and special teachers who have good expert knowledge and skills for preparing specific educational experiences for maximizing each student’s individual potential. Similar viewpoints have also been presented in the international literature: for example, Kauffman and Hornby (2020) criticized inclusive education ideology and leading scholars in the field for the unrealistic claims regarding its implementation and outcomes. They concluded as: “Appropriate instruction is by far the most important task of education for all students, including those with disabilities. Making appropriate instruction a reality for all students requires special education, including teachers with special training, rather than a generic, ‘one size fits all’ or all-purpose preparation” (p. 10).

### Inclusion for All

In contrast to voices arguing for creating different learning environments for different children, scholars, policy makers, teachers, and parents in favor of inclusion for all stress, in different talks and articles, that all children in a society should have an equal right to get adequate opportunities to develop wellbeing, agency, identities, and competences in order to become capable to participate fully and equally in the society (UNESCO, 1994; Ainscow and César, 2006; Cigman, 2007; Felder, 2019). This objective cannot be reached if some children are educated in a segregated context.

Inspired by social constructivist approaches to learning, teacher educators supporting inclusive education argue that child development depends not only on inherited capacities, but it is also constructed by shared social values, access to educational institutions, technologies (including assistive technologies), and other relevant social resources as well as quality of support provided to the child and opportunities to participate fully and equally in a community.

Teacher educators and policy makers would agree that it is true that current educational systems (schools, teachers, initial education of teachers, practices, technologies, teaching and learning materials, etc.) in many countries have been established based on an assumption that “regular” education, schools, and teachers should work only with “typical” children and other children need to be educated in a specially designed and segregated environment, that is, “special” education (Carrington, 1999; Croll and Moses, 2000; Dyson et al., 2002; Radó et al., 2016; Zgaga, 2019; Koutsouris et al., 2020). However, they would argue that in such an environment, children cannot develop a sense of belonging nor can become full members of the society because of marginalized status and limited opportunities to grow with others (Freeman and Alkin, 2000; Farrell, 2010; Koller et al., 2018). Moreover, in a special education, setting relationships, practices, and technologies tend to be adapted to their constraints instead of being designed to enable children to fully participate in education and society in spite of constraints. Similarly, parents, teachers, and kindergarten/school leaders favoring inclusive education in Estonia would argue for social justice ideas: the importance of growing up within the community and learning at a kindergarten/school close to their home. A father of a child with speech difficulties, who was contacted by an author of this article and asked why he favored his child attending a regular kindergarten instead of a specialized kindergarten, pointed out as: “I can’t distinguish my child, who has special (or rather specific) needs, from any other child. How can I agree with her being placed in a school which labels her directly and indirectly as a person who does not fit the norm? Especially when attending kindergarten, she is as special and as normal as every other child who she plays with and a child who plays with her. This should be the norm for any healthy development of a child.” Similarly, a teacher and master student (Konetski-Ramul, 2021) and a head of support specialists services (Labi, 2019) have argued for inclusive education in articles published in the national teachers’ newspaper in Estonia. In these articles, the authors urged for not separating students with special needs easily to special classes or special schools, e.g., Labi (2019) pointed out as: “If today we separate one quarter of children for fear that their involvement could negatively affect the well-being of the other three quarters of children, then as adults there are people in the labor market, in families, or even in the queue at the store, who cannot cope with each other. It is more sustainable to grow together, learn from each other and cope with each other throughout the school journey.” Many parents of special needs children would also argue that the most important goal for them is for their children to adapt to society and learn to live with other people. To illustrate this idea, a mother of a young child with multiple disabilities pointed out during a public speech in Estonia that her family’s “goal is to support him so he would become a taxpayer.”

In order to have an equal opportunity, all children need to be educated in regular education that have conditions, capacities, and resources to be able to adapt to the children needs, capacities, and constraints. Following this, in a case when a school, teachers, discourses, practices, and technologies are not aligned with the needs of some students, it cannot be an acceptable reason for the exclusion of the child, but for adapting the education to the child and his/her learning and developmental needs (Farrell, 2010; Arcidiacono and Baucal, 2020).

The majority of Estonian teachers has adopted learner-centered views about education as reported in international comparison studies, such as TALIS 2013, 2018, and a smaller group has also learned to implement these in practice (many Estonian teachers are still rather traditional and subject-oriented in their teaching practice; OECD, 2014, 2020; see also Leijen and Pedaste, 2018). Teachers who have accepted the child-centered view might not consider a class as a unified mass, instead they might perceive children anyway as special and different, notice variety, individual differences and adapt their teaching accordingly (Breeman et al., 2015). Following, adapting their teaching for a child with special needs would not be so different from any other adaptation of teaching for the child’s needs and interests. While discussing the possible challenges of inclusive education during an in-service course taught by the first author of the paper in autumn 2019, a teacher pointed out that “it is very interesting and positively challenging to teach a group of students with a large variety. These are (my) favorite classes.” This indicates that teachers might find diversity and variety enriching for themselves as professionals.

## Goal of the Paper

The aim of this paper is to show, through the conceptual analysis of the two above-mentioned discourses, that it is possible to put these two narratives in a dialogical relation to identify their contribution to position children (with and without special needs) and teachers with respect to the voices they promote or silence about inclusive education, the power relations they constitute, and the values and practices they enact or prevent.

## Materials and Methods

We propose an analytical approach based on the argumentum model of topics (AMT) that aims at systematically reconstructing the inferential configuration of arguments; namely, the deep structure of reasoning underlying the connection between a standpoint and the argument(s) in its support (Rigotti and Greco Morasso, 2009). The general principle underlying the reconstruction of the inferential configuration of argumentation is that of finding those implicit premises that are necessary for the argumentation to be valid.

In the AMT, two fundamental components should be distinguished when bringing to light the inferential relation binding the premises to the conclusion of an argumentation. First, an argument envisages a topical component, which focuses on the inferential connection activated by the argument, corresponding to the abstract reasoning that justifies the passage from the premises (arguments) to the conclusion (standpoint). The inferential connection underlying the argument is named with the traditional term maxim. Maxims are inferential connections generated by a certain semantic ontological domain named locus. Second, an endoxical component, which consists of the implicit or explicit material premises shared by the discussants that, combined with the topical component, ground the standpoint. These premises include endoxa, i.e., general principles, values, and assumptions that typically belong to the specific context, and data, basically coinciding with punctual information and facts regarding the specific situation at hand and usually representing the part of the argument that is made explicit in the text (Rigotti and Greco Morasso, 2011). Despite its particular concern for the inferential aspects of argumentation, the AMT accounts not only for the logical aspects of the argumentative exchange, but also for its embeddedness in the parties’ relationship, and thus proves to be particularly suited for the argumentative analysis of public discourses.

In the present paper, we refer to the AMT to reconstruct the inferential structure of some arguments proposed by the two above-mentioned discourses, i.e., the type of reasoning underneath the arguments. In this sense, the model is assumed to be a guiding framework for the analysis, since it provides the criteria for the investigation of argumentative positions and for the identification of different components of each discourse. It is used to highlight points of contention and dialogue, as well as the explicit and implicit arguments advanced by the involved sustainers of the two narratives. The application of this analytic method in the study of public discourses, such as the role of inclusive education, is assumed to reinforce the possibilities of understanding how people discursively position themselves as involved partners in the management of the selected issue, namely, inclusive education.

## Results

According to the AMT, the following analytical components must be identified as: the maxim on which the argumentation is based and the relative locus at work; the endoxon, i.e., the premises shared by the discussants, and the datum, i.e., the punctual information and facts regarding the specific situation at hand (usually representing the part of the argument that is made explicit in the text) to which the argument is linked. The results of the AMT’s reconstruction will be represented through graphical tools adopted to show the above-mentioned components.

Generally speaking, the different arguments used by the parties can be viewed in terms of a constellation of features (Goodwin, 2006), including various interactional structures connected to aims, perceptions, directives, accounts, etc. In the present paper, we will limit our conceptual analysis of two narratives to some elements that are essential for the aim of the study, although we are aware that this is a partial choice. Accordingly, the locus at work for the maxims will not appear in our schemes and only the arguments sustaining the main ideal view of each narrative and the presumed positions associated with the selected arguments will be presented.

In the next sections, we propose two examples of AMT based on selected arguments for each discourse.

### Reconstructing the Inferential Structure of the First Discourse Argument

The first discourse (“inclusion for some”) proposes as a standpoint that students with special needs require specialized educational settings. The argument advanced to sustain this position is that specialized settings are accommodating to the student’s capacities and needs.

**Figure 1:**
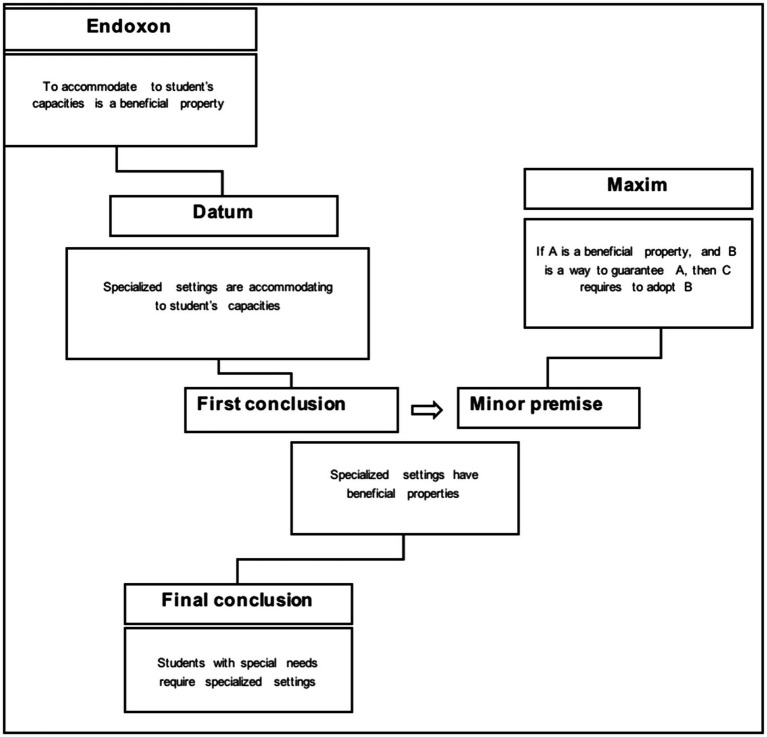
AMT-based reconstruction of the first discourse argument.

Figure 1 shows the representation of such an argument based on the AMT. On the right hand of the diagram, the inferential principle, i.e., the maxim, on which the argumentation is based is specified as: “to provide a beneficial property to the student, it is required to adopt a system that guarantees this beneficial property.” The AMT representation allows consideration of the contextual premises that are implicitly or explicitly used in argumentation. This may be found on the left hand of the diagram, where a second line of reasoning is developed that supports the former one. This is why the first conclusion on the left becomes the minor premise on the right. In this way, the crossing of contextual and formal premises that is characteristic of argumentation is accounted for in the AMT. The endoxon refers in this case to common knowledge about the main idea of the accommodation principles: “To accommodate to the student’s capacities is a beneficial property.” The datum (“Specialized settings are accommodating to student capacities”) combined with the endoxon produces the conclusion that “Specialized settings have beneficial properties.”

In the first discourse, if the accommodation is considered beneficial for a student with special needs, and if specialized settings are recognized as environments that can guarantee a process of accommodation, then it is valuable to require that students with special needs should be placed in specialized settings.

### Reconstructing the Inferential Structure of the Second Discourse Argument

**Figure 2:**
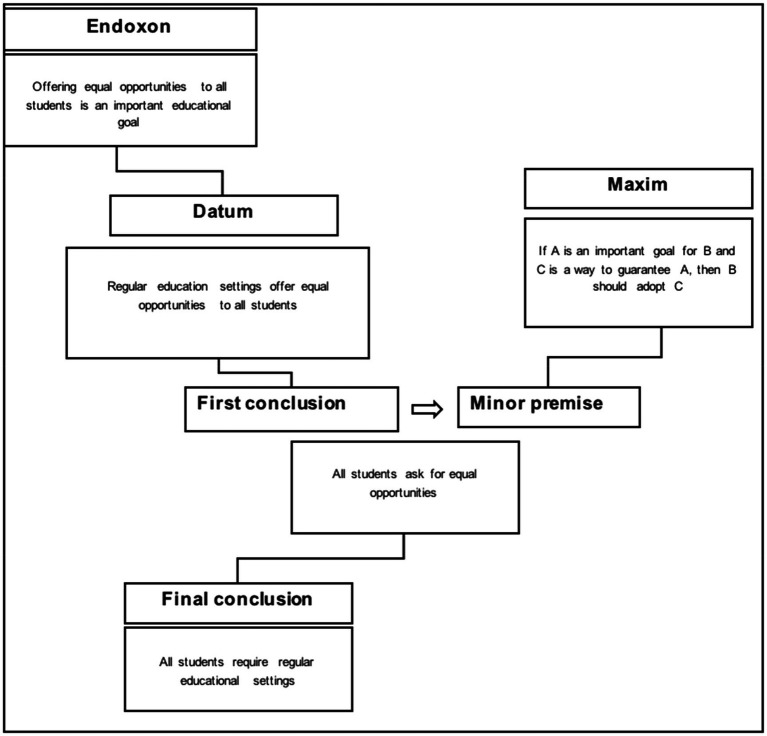
AMT-based reconstruction of the second argument.

The second discourse (“inclusion for all”) proposes as a standpoint that all students require regular educational settings. The argument advanced to sustain this position should be summarized as follows: Regular settings offer equal opportunities to all students. Figure 2 shows the representation of such an argument based on the AMT.

On the right hand of the diagram, the inferential principle, i.e., the maxim, on which the argumentation is based is specified as: “if the offer of equal opportunities is an important educational goal, and there is a way to guarantee such a goal, then this way should be adopted.” Concerning the contextual premises that are implicitly or explicitly used in argumentation, the endoxon refers to common knowledge about the main idea of the educational goals: “Education should offer equal opportunities to all students.” The datum (“Regular settings offer equal opportunities to all students”) combined with the endoxon produces the conclusion that “All students require regular educational settings.”

The discourse indicates that if offering equal opportunities to all students (by exposing them to similar conditions) are considered an important educational goal, and if regular settings are recognized as environments that can guarantee to offer equal opportunities, then it is valuable to require that all students be placed in regular settings.

### Implicit in Two Discourses

The models referring to the two discourses about inclusive education are showing in both cases reasonable arguments advanced to sustain the positions and the perspectives they intend to promote. For each discourse, accountable elements are proposed to show the pertinence of the approach and to sustain the idea of education that is considered as adequate for society.

The two discourses position the children as the main key-players in the educational endeavor: In fact, inclusive education should sustain the requirement for appropriate settings (special and regular) that are able to allow students (with and without special needs) to develop their capacities and to become members of the society. In this sense, the two discourses share a similar preoccupation and aim to play in the service of children’s development. However, it is also true that both discourses promote reasons that seem to position the children within different frames, for example, in terms of temporality. In fact, the first discourse (“inclusion for some”) focuses on the need to guarantee a process of accommodation to the children’s needs in order to guarantee a system that allows students to develop their capacities. In this sense, a short-term perspective is promoted, because the goal behind the sustained discourse is to be able to act adequately in the “here and now” of the contingent situations. By contrast, the second discourse (“inclusion for all”) advances the idea that offering equal opportunities to all students constitute the main goal of education. In this sense, a long-term perspective is promoted in terms of capacity to ensure the conditions that will guarantee the future realization of students as full members of the society. These elements, connecting the two discourses along a temporal dimension, will be discussed in the next section of the paper, as well as the implications in terms of positions that children and teachers should take according to their voice, the power relations that are connected to this, and the values that are enacted or prevented.

## Discussion

Although we have identified these two discourses in the current ongoing debate on inclusive education in Estonia, they encapsulated long lasting conflicting positions that can be recognized in many countries and communities. Moreover, these discourses also reflect political, policy, cultural, and identity “wars” that are present in many countries since the Salamanca Statement (UNESCO, 1994), calling societies to put forward the inclusive education on their education policy agenda. The “war” on inclusive education is related to the fact that educational policies are inherently political, since they always involve values, interests, power games, choices, prioritization, and allocation of resources (Barton, 1997). Moreover, different sides propose different values and ideals; that is, they postulate different desired outcomes and different visions of the future citizen (Magnússon, 2019). However, it is worth noting that current conflicting debates are just another step in an historical process of a struggle of regular schools between exclusion and inclusion of children and youth perceived and treated as different from dominant groups, in relation to various characteristics, such as socioeconomic status, gender, and race (Boroson, 2017). In fact, the meaningful inclusion of individuals who are different from the majority has been fraught in many ways. The evolution of educational systems with respect to the inclusion of students who are different in terms of race, gender, or ability was considered to be of questionable worth, an obstacle on teachers’ time and a threat to the status quo (West, 2000). Although today education in mainstream schools is guaranteed (Snyder et al., 2016), many educators and families still have a concern or even fear the “intrusion” into general education classrooms of students who are different than majority in terms of personal characteristics (physical, socioemotional, or cognitive) or ethnic, cultural, and socioeconomic background (Boroson, 2017). By considering the two discourses highlighted in this paper, we can consider that, on the one hand, those who are pro-specialist settings would argue that segregation works in favor of child with special education needs; on the other hand, those who view inclusion as a social justice issue might consider specialist settings as segregating like other forms of educational and historical segregation (e.g., gender and faith).

Two conclusions could be made from this. First, as with previous inclusion “wars,” the current one will be resolved when conflicting sides will manage to dialogize their conflicting positions. The second conclusion is that the current “war” is just an episode in a continuous historical story on social inclusion, so after that one, there will be some new inclusion “war” that might not be imaginable from the perspective of our current experience and knowledge. Having said that, our main objective is to identify their frameworks in terms of assumptions, power relationships, voices, rights, and values, as well as priorities and practices in order to propose a bridging between them and to dialogize current relation that is dominantly conflicting.

As it is already said, both discourses put a stress on children’s needs and recognize the duty of the education system to provide adequate conditions for their education. However, there is also an important difference in relation to the position and rights of children with special needs. The “inclusion for some” discourse recognizes the rights of children with special needs, but at the same time, it advocates that their rights need to be limited by practical constraints related to the implementation of the full inclusion in the regular school. In this way, this discourse gives a voice to children with special needs, but also to educational practitioners who are in many occasions not competent enough nor have adequate conditions and resources to ensure quality education to children with special needs in regular schools. Although both voices are represented in the discourse “inclusion for some,” it prioritizes somewhat the voice of educational practitioners. On the other hand, the “inclusion for all” discourse privileges the voice of children with special needs and their rights that need to be served by the society in the same way as the rights of all other citizens. It also recognizes practical and policy constraints at the level of the education system, schools, and practitioners, but it does not position their voices and concerns at the same level as the rights of children with special needs. Thus, it advocates that schools and practitioners ought to be equipped by adequate policies, training, and resources to be capable of serving the rights of children with special needs for the quality education in inclusive conditions.

Difference in prioritization of voices is related to the difference in basic values and distribution of power. The discourse “inclusion for some” suggests that the current potential of the education system, schools, and teachers should be put at the first place and that rights of children with special needs should be realized progressively following the improvement of the potential of the education system to ensure high quality inclusive education. In this way, it gives more power to the majority, to the education system, and practitioners since it calls that rights on quality education need to be aligned with the potential of the education system to serve this right. However, in this way, it also creates an opportunity for using current lack of capacities in regular schools for ensuring inclusive education as a reason for postponing the realization of rights of children with special needs. If for some reason there is no political will or if the majority of educational practitioners is not willing to transform their beliefs, competences, and practices, then it might effectively maintain current conditions for some time (potentially endlessly). On the other hand, the “inclusion for all” discourse privileges the right of children with special needs over current conditions and lack of capacities and resources advocating that the latter needs to be transformed as quickly as possible. Consequently, it places higher power to the children with special needs and their fundamental rights than to eventual practical and political constraints of various kinds. Nevertheless, it might be related to some unintended negative consequence in the implementation of inclusive education. Forcing a full implementation of inclusive education when regular schools and practitioners are not prepared adequately might result in various negative consequences. These consequences might be counterproductive in terms of defending rights of children with special needs and effectively postpone the implementation of inclusive education. Therefore, in spite of differences in terms of basic values and power relations putting forward in two discourses, it is possible to identify a common interest. It is related to the successful implementation of inclusive education and the minimization of risk both for children with special needs and for education practitioners and schools including children without special needs.

Concerning the implementation of inclusive education, there are two opposite perspectives creating a major conflict between the two discourses. Being grounded on previous founding ideas, the “inclusion for some” advocates for some form of special education provision mostly in separate and specialized schools, while the “inclusion for all” discourse stands up for desegregation and full inclusion of children with special needs in regular schools. According to UNESCO (2020), the implications in developing forms of education that are effective for all children are related to three levels: educational (to develop ways of teaching that respond to individual differences and that therefore benefit all children), social (to change attitudes to difference by educating all children within a non-discriminatory society), and economic (it is likely to be less costly to establish and maintain schools).

These positions reflect their difference in terms of future priorities (Ydo, 2020). The “inclusion for some” discourse is focused to optimize provision of education as an ultimate goal. Hence, it prefers providing education in a specialized environment since it enables full accommodation to specific educational needs of children attending special schools. In this way, children with special needs might have best opportunities to learn in their way and to achieve education goals. On the other hand, the discourse “inclusion for all” calls for a more comprehensive ultimate goal. These goals ought to be to empower and enable children with special needs to become active citizens who will participate fully and equally in the society and to pursue their own life projects. Projecting this ultimate goal for education of children with special needs, the discourse “inclusion for all” pursues a full inclusion in regular schools since education in segregated institutions prevents children with special needs from becoming full members of the society. This difference in terms of the ultimate goal of education of children with special needs might seem as unresolvable. It also can make sense why the “war” between the two discourses and the communities organized upon them is very frequently concentrated on the special school issue. However, in our view, this opposition might be bridged by relating the two discourses to different time perspectives (as it has been already mentioned earlier). The common ground might be that all children with special needs are fully included in regular schools in order to enable and empower them to become active and equal future citizens, but to keep special schools and special education teachers as additional resources where different students from regular schools can get different forms of supplementary support according to their needs occasionally or in a longer period. This approach would require establishing a good and productive professional collaboration between regular and special schools as resource centers, as well as between teachers from regular schools and special education teachers. Based on a good professional collaboration and complementary professional competences of all teachers (including special education teachers), children with special needs would get an additional support during classes in regular schools or when it is needed in a special school (for example, when the child needs a specialized treatment or to get additional training for using some assistive technology). It is true that this arrangement could be challenged by some practical issues and would require a modification of regular institutional organization and practices. However, it would improve opportunities for children with special needs to become competent future citizens, and for the education system and the society to become inclusive.

Furthermore, additional common ground might be related to the pace of the long-term implementation of inclusive education. The discourse “inclusion for all” provides a strong argument why inclusive education is the principal way to empower and enable children with special needs to grow up with a feeling that they are equal members of the society and with a dignity to take part fully in the life of the community so to pursue their life projects and contribute to the society. However, the discourse “inclusion for some” pinpoints in a good way that journey toward the ultimate goal cannot be straightforward nor quick because it is related to the transformative potential of the society and the education system imposing important constraints. Although these constraints are malleable and temporary, they need to be addressed in any implementation plan for inclusive education. Therefore, we assume that the two discourses can be bridged in the sense that one of them crystalizes and advocates what ought to be long-term goals for the implementation of inclusive education, while the second one articulates practical constraints and barriers that need to be overcome in order to make inclusive education real.

## Conclusion

In this paper, we utilized the AMT for analyzing two somewhat opposing discourses regarding inclusive education, namely, the “inclusion for some” and “inclusion for all.” We reconstructed the inferential configurations of the arguments of each narrative, identified how the two definitions contribute to position children with and without special needs and their teachers. The results showed several similarities and differences between the discourses. We also identified some possibilities to bridge the two narratives; most importantly, by relating to different time perspectives, these two discourses stress: “Inclusion for some,” which tends to focus on the present situation and attending to the particularities of the child, is valuable for realizing the long-term and sometimes idealistic goals of “inclusion for all” and *vice versa*, “inclusion for all,” which stresses participation and learning with peers, is beneficial for realizing the goals of “inclusion for some” – to maximize each child’s potential in real life – since regular schools resemble society more closely than segregated schools. Productive professional collaboration between different parties is required to realize both visions of inclusive education. We also suggest further investigations to deepen this research line in the future, through face-to-face interviews with politicians, school managers, teachers, and parents who could better delineate the different positions according to their role and involvement with children with special needs.

## Author Contributions

All the authors originated the paper’s ideas and designed the conceptual analysis. ÄL was engaged in presenting the context of the study, as well as interpreting and discussing the findings and writing the conclusion. FA focused on the method, analysis, and interpretation of the narratives. AB focused mainly on interpreting and discussing the findings. All authors approved the final version of the manuscript to be published.

## Funding

This research was conducted with financial support by the European Regional Development Fund under Grant Enhancement of Research and Development Capacity of Teacher Education Competence Centre Pedagogicum [grant no. NSVHI19074], as well as the contribution of the Research Department, University of Teacher Education BEJUNE (Switzerland).

## Conflict of Interest

The authors declare that the research was conducted in the absence of any commercial or financial relationships that could be construed as a potential conflict of interest.

## Publisher’s Note

All claims expressed in this article are solely those of the authors and do not necessarily represent those of their affiliated organizations, or those of the publisher, the editors and the reviewers. Any product that may be evaluated in this article, or claim that may be made by its manufacturer, is not guaranteed or endorsed by the publisher.

## Footnotes

^1^In line with OECD definition, in this paper, we refer to “children with special education needs” as an umbrella term indicating children who require additional resources or accommodating dominant educational practices in order to ensure equal learning opportunities to them. The term refers to children with disabilities, children with learning difficulties, and children living in disadvantage conditions (OECD, 2007).
